# The role of deubiquitinases in cardiac disease

**DOI:** 10.1017/erm.2024.2

**Published:** 2024-03-25

**Authors:** Xiaona Zhan, Yi Yang, Qing Li, Fan He

**Affiliations:** Department of Nephrology, Tongji Hospital Affiliated to Tongji Medical College, Huazhong University of Science and Technology, Wuhan 430030, People's Republic of China

**Keywords:** cardiac disease, cardiac fibrosis, cardiac hypertrophy, deubiquitinases, myocardial ischaemia-reperfusion injury, myocarditis

## Abstract

Deubiquitinases are a group of proteins that identify and digest monoubiquitin chains or polyubiquitin chains attached to substrate proteins, preventing the substrate protein from being degraded by the ubiquitin-proteasome system. Deubiquitinases regulate cellular autophagy, metabolism and oxidative stress by acting on different substrate proteins. Recent studies have revealed that deubiquitinases act as a critical regulator in various cardiac diseases, and control the onset and progression of cardiac disease through a board range of mechanism. This review summarizes the function of different deubiquitinases in cardiac disease, including cardiac hypertrophy, myocardial infarction and diabetes mellitus-related cardiac disease. Besides, this review briefly recapitulates the role of deubiquitinases modulators in cardiac disease, providing the potential therapeutic targets in the future.

## Introduction

Protein synthesis and degradation must be in balance for cellular proteins to remain stable (Ref. 1). The ubiquitin-proteasome system primarily controls the majority of protein degradation in eukaryotic cells (Ref. 2). Ubiquitin is a polypeptide composed of 76 highly conserved amino acids (Ref. 3). Ubiquitination is the process of connecting ubiquitin with protein to form the ubiquitin chains under the action of ubiquitin ligase. Ubiquitination regulates a variety of biological functions, the most important of which is protein degradation mediated by 26S proteasome (Ref. 4). Ubiquitination is a reversible process, which is strictly regulated. Deubiquitination is the main negative regulation process. It recognizes and cleaves the ubiquitin chains of substrate protein through a series of deubiquitinases, thus inhibiting the degradation process of protein (Ref. 5). The balance between ubiquitination and deubiquitination is crucial in the human body, regulating a variety of diseases such as cancers (Ref. 6), neurodegenerative diseases (Ref. 7) and regulating immune cells (Ref. 8).

Recently, more and more research has confirmed that deubiquitinases represent a pivotal regulator in cardiac disease by recognizing different substrate proteins and regulating a variety of signalling pathways and mechanisms (Refs 9, 10). However, until now, there are few reviews on the regulatory role of deubiquitinases in cardiac disease. This review summarizes the recent basic research on the function of deubiquitinases in cardiac disease, specifically introduces the emerging roles of deubiquitinases in regulating cardiac diseases and their specific molecular mechanism, and provides a new strategy for the therapy of cardiac disease in the future.

## Ubiquitin proteasome system

Since it was initially identified in the 1970s, ubiquitin has been found in a wide variety of creatures (Ref. 11). Ubiquitin activating enzyme (E1) joins the lysine residue at the C-terminal of ubiquitin to its own cysteine residue during the ubiquitination process by hydrolysing ATP. After the ubiquitin has been delivered to ubiquitin coupling enzyme (E2), the target protein is then joined with ubiquitin by the ubiquitin ligase (E3) (Ref. 12). The majority of proteins in the human body can be marked with ubiquitin, and the 26S proteasome can identify and degrade ubiquitinated proteins. There are many different forms of ubiquitination, but the three most prevalent types are monoubiquitination, K48-linked and K63-linked polyubiquitination (Ref. 13). After the substrate protein is modified by ubiquitination, different types of ubiquitination will lead to different protein fate. Monoubiquitination is associated with protein recognition or allosteric regulation, while K48-linked and K63-linked polyubiquitination may lead to protein degradation and signal transduction, respectively (Ref. 5).

As one of the most important post-translational modifications, ubiquitination not only plays an important role in regulating protein degradation, but also plays a key role in regulating cell cycle (Ref. 14), antigen presentation (Ref. 15), immune signal transduction (Ref. 16), transcription regulation and apoptosis (Ref. 17).

## Deubiquitinases

Deubiquitination refers to the process of recognizing ubiquitin-labelled proteins, hydrolysing and removing ubiquitin chains on some amino acid residues of substrate proteins under the action of a series of deubiquitinases (Ref. 18). Deubiquitinases stabilize proteins to maintain their functions by reversing the ubiquitination process. Deubiquitinases can be divided into six families: the ubiquitin-specific proteases (USPs), the ovarian tumour-related proteases (OTUs), the ubiquitin C-terminal hydrolases (UCHs), Machado Joseph disease proteins (MJDs), Mindys family motif interacting with ubiquitin containing novel dub family (MINDYs) and JAB1/MPN/MOV34 metalloproteinases (JAMMs). The first five deubiquitinases belong to cysteine protease (Ref. 19). The USPs family is the family with the most members in the deubiquitinases, which can recognize the ubiquitin chains linked by K48, K63 and MET1. The protease of the MJD family tends to recognize the K48-linked and K63-linked polyubiquitination, the protease of JAMMs tends to recognize the K63-linked polyubiquitination, and MINDYs tend to recognize and hydrolyse the K48-linked polyubiquitination from the distal end (Ref. 5).

Deubiquitinases may either deubiquitinate a broad area surrounding the hydrophobic region of ubiquitin or they can precisely remove ubiquitin by identifying and hydrolysing the ester bond, peptide bond or isopeptide bond at the carboxyl terminus of ubiquitin (Ref. 19). Deubiquitinases can also inhibit the function of E3 ligase by hydrolysing the peptide linkage that connects the target protein's lysine residue to the C-terminal of ubiquitin (Ref. 20). Deubiquitinases vary in number depending on the species, and there are roughly 100 different types in human cells (Ref. 21). Numerous studies have demonstrated that deubiquitinases can regulate cell growth and development, mediate physiological signalling pathways and maintain cell homeostasis (Ref. 22). Moreover, deubiquitinases also exert a significant regulatory influence over the pathophysiological processes of a number of diseases, including tumours (Ref. 23), inflammatory bowel disease (Ref. 24) and vascular disease (Ref. 25).

## Deubiquitinase is an essential regulator of cardiac physiology and homeostasis

USP17 subfamily is comprised of USP17k, USP17l, USP17m and USP17n. Researchers discovered that the USP17 subfamily members were significantly expressed in human myocardial tissue, indicating that the USP17 family may be crucial in preserving the physiological function of the heart (Ref. 26). Additionally, DUB-1a, a newly discovered deubiquitinase subfamily member, was discovered in lymphocytes for the first time. Researchers provided experimental evidence that DUB-1a was also expressed in the mouse heart, suggesting that it may be involved in preserving cardiac homeostasis (Ref. 27).

A critical role of cylindromatosis (CYLD) in maintaining subcellular region was supported by research in which CYLD promoted the stability of plakoglobin by reducing its K63-linked ubiquitin chains. Plakoglobin is an intermediate protein that stabilizes the gap junction of myocardial cells. The deubiquitination of plakoglobin stabilized its connection with desmoplakin, contributing to the transmission of Cx43 to the inserted intervertebral disc. The stable existence of CYLD is crucial for the development and maturation of myocardial cells (Ref. 28).

## Role of deubiquitinases in cardiac disease

### Cardiac hypertrophy

**Figure 1.**
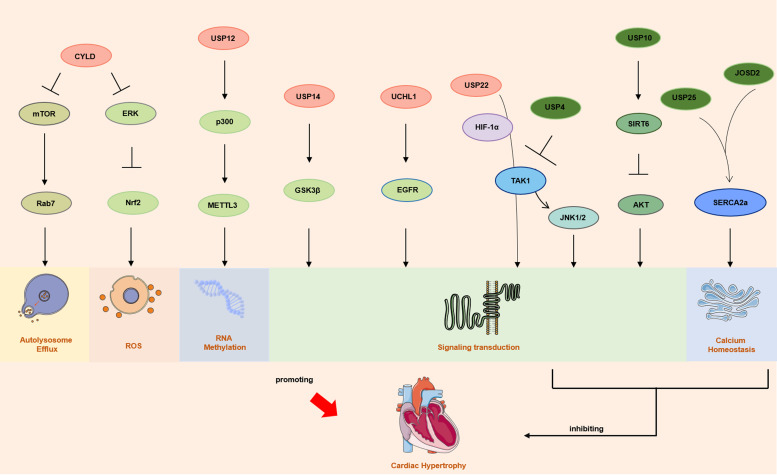
The main deubiquitinases involved in the pathogenesis of cardiac hypertrophy. *Notes*: Multiple deubiquitinases regulate cardiac hypertrophy through mechanisms such as autophagy, ROS, RNA methylation, signal transduction, and calcium homeostasis. CYLD: cylindromatosis; mTOR: mammalian target of rapamycin; Rab7: Ras-related protein Rab-7a; Erk: extracellular signal-regulated kinase; Nrf2: nuclear factor erythroid-2-related factor 2; USP: ubiquitin-specific protease; METTL3: metallothionein-like 3; GSK3β: Glycogen synthase kinase 3β; UCHL1: ubiquitin C-terminal hydrolases L1; EGFR: epidermal growth factor receptor; HIF-1α: hypoxia inducible factor-1α; TAK1: transforming growth factor-β-activated kinase 1; JNK1/2: c-Jun N-terminal kinase 1/2; SIRT6: sirtuin 6; AKT: protein kinase B; JOSD2: josephin domain-containing protein 2; SERCA2a: sarco/endoplasmic reticulum Ca_2+_-ATPase.

The protein kinase pathway (PI3K/Akt signalling pathway, cGMP/PKG signalling pathway, etc.), calcium-mediated signalling pathway (CaMKⅡ signalling pathway, CaN-NFAT signalling pathway, etc.) and Wnt signalling pathway are the main signalling pathways currently involved in pathological myocardial hypertrophy (Refs 29, 30). Numerous studies have shown that deubiquitinases modulate the development of cardiac hypertrophy by regulating different signalling pathways that are associated with cell inflammation (Ref. 31), apoptosis (Ref. 32), autophagy (Ref. 33), mitochondrial homeostasis (Ref. 34) and metabolic changes (Ref. 35) (Fig. 1).

Previous studies have found that the expression profile of USP has changed in hypertrophic hearts, which indicated that USP may be a biomarker of cardiac hypertrophy or a regulator in the progression of cardiac remodelling. In the chronic heart failure model, the mRNA expression of USP19 was significantly downregulated in chronic heart failure models (Ref. 36). In addition, by comparing the GEO dataset and the mRNA level of USP2 in hypertrophic heart induced by trans-arterial coarctation (TAC), it was determined that the expression of USP2 was downregulated in hypertrophic heart, and the overexpression of USP2 could relieve the cardiac hypertrophy induced by TAC. However, further research was still warranted to explore the molecular mechanism of USP2 in myocardial hypertrophy caused by pressure overload (Ref. 37).

Transforming growth factor-activated kinase 1 (TAK1) is a MEKK family member and the upstream of JNK1/2 and p38 (Refs 38, 39). Overexpression of TAK1 could promote the activation of NF-*κ*B and NFAT signalling pathways, and upregulate the expression of cardiac hypertrophy markers (Ref. 40). In the hearts of patients with heart failure and hypertrophic rodent hearts, the mRNA and protein expression of USP4 was downregulated, while overexpression of USP4 could alleviate cardiac hypertrophy both in vivo and in vitro. USP4 played an anti-myocardial hypertrophy effect in regulating pathological myocardial hypertrophy and fibrosis by blocking the activation of the TAK1-(JNK1/2)/p38 signalling pathway (Ref. 41). However, unlike the anti-hypertrophic effect of USP4, USP22 exerted its pro myocardial hypertrophy effect by activating the TAK1 signalling pathway. USP22 stabilized the hypoxia-inducible factor-1*α* (HIF-1*α*) protein through its deubiquitinase activity, activating the TAK1 signalling pathway and exacerbating myocardial hypertrophy-induced pressure overload (Ref. 42).

GSK-3*β*, as a widely expressed serine threonine protein kinase, can phosphorylate different substrates and act as a significant negative regulator in regulating cardiac development and myocardial hypertrophy by activating a wide range of downstream signalling molecules (Ref. 43). In a variety of rodent models of cardiac hypertrophy, the specific overexpression of GSK-3*β* in myocardium could significantly alleviate myocardial hypertrophy and fibrosis by reducing the nuclear localization of NFAT (Refs 44, 45). The phosphorylation of GSK-3*β* is bidirectional. The phosphorylation of the C-terminal Tyr216 site can activate GSK-3*β* (Ref. 46), while the phosphorylation of C-terminal Ser389 and Thr390 sites leads to the inactivation of GSK-3*β* (Ref. 47). USP14 has been shown to upregulate the phosphorylation of GSK-3*β*. In the models of myocardial hypertrophy induced by abdominal aortic constriction (AAC) and Ang II, USP14 accelerated the development of cardiac hypertrophy and worsen cardiac function by activating GSK-3*β*. However, it is not clear whether USP14 directly combined with GSK-3*β*, or indirectly interacted with GSK-3*β* through other molecules. Further experimental research is needed to investigate the interaction between USP14 and GSK-3*β* and the phosphorylation site of GSK-3*β* (Ref. 48).

In addition to the typical signalling pathways, skeletal muscle LIM protein 1 (SLIM1) is a key factor related to the pathogenesis of myopathy and cardiomyopathy (Refs 49, 50). A study on USP15 transgenic mice showed that the upregulation of USP15 exacerbated myocardial hypertrophy by interacting with the SLIM1 protein, cutting the SLIM1 ubiquitin chains and stabilizing the SLIM1 protein. Additionally, in the cardiac tissue of USP15 transgenic mice, the mRNA level of SLIM1 was also higher than that of WT mice, which indicated that USP15 may upregulate the expression of SLIM1 both at the transcriptional level and post-translational modification level (Ref. 51).

The calcium homeostasis of cardiomyocyte is indispensable for the maintenance of cardiac function (Refs 52, 53). Sarco/endoplasmic reticulum Ca^2+^-ATPase (SERCA2a), located on the sarcoplasmic reticulum, is regarded as a key protein for maintaining calcium homeostasis, and multiple studies have shown that there are several post translational modification sites in SERCA2a (Ref. 54). Research showed that USP25, as a protective protein of the heart, played an anti-hypertrophic role in pathological myocardial hypertrophy (Ref. 55). Ye *et al.* found that knocking out USP25 in mice exacerbated myocardial hypertrophy induced by angiotensin II and TAC. USP25 exerted its anti-hypertrophic effect by regulating the SERCA2a. USP25 stabilized intracellular calcium homeostasis and alleviated myocardial hypertrophy by removing the K48-linked ubiquitin chains of SERCA2a protein (Ref. 55). Coincidentally, the team also found that JOSD2 maintained myocardial calcium homeostasis by upregulating SERCA2a, thereby attenuating cardiac hypertrophy (Ref. 56). However, there are currently no agonists or analogues for USP25 or JOSD2, and more research is needed to promote their clinical conversion.

The sirtuin protein family (SIRT family), a highly conserved protein, is a kind of nicotinamide adenine dinucleotide (NAD^+^) dependent deacetylase. Among the SIRT family members, SIRT6 has been proved in multiple studies to regulate the NF-*κ*B signalling pathway or the Akt signalling pathway, both of which were connected to the development of cardiac hypertrophy (Ref. 57). Zhang *et al.* proved that USP10 exerted its anti-hypertrophic effect mainly by inhibiting the Akt signalling pathway, rather than through the common downstream signalling pathways of USP10 (such as p53, AMPK, TRAF6). Further studies showed that SIRT6 acted as an intermediate messenger of USP10 inhibiting the Akt signalling pathway. USP10 bound and stabilized SIRT6 by inhibiting the activation of Akt/GSK3*β* and mTOR/p70S6k signalling pathway, resulting in the remission of myocardial hypertrophy and fibrosis (Ref. 10). Aside from SIRT6, SIRT2 also contributes to the occurrence and progression of cardiac hypertrophy. SIRT2-deficient mice develop spontaneous pathological cardiac hypertrophy through LKB1/AMPK (Ref. 58). Mei *et al.* discovered that the mRNA and protein level of CSN6 was upregulated both in the Ang II-induced hypertrophic cardiomyocyte and hypertrophic cardiac tissue. The researchers pointed out that inhibiting the expression of CSN6 might relieve the progression of cardiac hypertrophy. Additional research revealed that CSN6 could contribute to cardiac hypertrophy by stabilizing Nkx2 and removing the K48-linked ubiquitin chains of Nkx2.2. Nkx2.2, a common transcription factor, attached to the promoter of SIRT2 and prevented the activation of transcription. CSN6 stabilized Nkx2.2, which blocked the transcription of SIRT2, resulting in ventricular hypertrophy and heart failure (Ref. 59).

Signal transducer and activator of transcription 3 (STAT3) is a protein that is involved in both cytoplasmic signal transduction and nuclear transcriptional activation. It is one of the key factors exacerbating myocardial hypertrophy and is regulated by deubiquitinase. Wang *et al*. demonstrated that OTUD1 promoted the development of pathological myocardial hypertrophy by removing the K63-linked ubiquitin chains of STAT3 and stabilizing the protein in various rodent models of myocardial hypertrophy. This study further elucidated that OTUD1 bound to the SH2 domain of STAT3 through its cysteine site at position 320, thereby exerting the effect of deubiquitination (Ref. 60).

In addition to transcription factors, at the level of gene regulation, methyltransferases in cardiomyocytes are also indirectly regulated by deubiquitinases. USP12 deubiquitinated p300 to activate the transcription of methyltransferase-like 3 (METTL3), which resulted in the abnormal m6A RNA methylation in cardiomyocyte and exacerbated cardiac hypertrophy (Ref. 61).

Even though the function of autophagy in the heart is still debatable, recent studies tend to view the activation of autophagy as a protective process of cardiac hypertrophy (Ref. 62). Autophagy is regulated by several kinds of deubiquitinases, including CYLD (Refs 63, 64, 65). An experiment has demonstrated that CYLD could give rise to cardiac hypertrophy by mediating autophagy in cardiac hypertrophy induced by pressure overload. Specifically, compared with wild-type mice with TAC-induced cardiac hypertrophy, autophagic flow in cardiac myocytes was significantly blocked in the hypertrophic heart of mice with cardiomyocyte-specific CYLD overexpression, resulting in cardiac morphological changes and dysfunction in transgenic mice (Ref. 66). The role of CYLD was only related to the restricted autolysosome efflux but did not affect basic autophagy and the combination of autophagosome and lysosome. Further study on the downstream molecules of CYLD revealed that the hypertrophic effect of CYLD was partly attributed to the inactivation of mTORC1 and the upregulation of Rab7, which was important in the excretion of autolysates. Another study provided another potential molecular mechanism of CYLD in pressure load-induced cardiac hypertrophy. CYLD blocked Nrf2-mediated antioxidant capacity by inhibiting Erk, p38/AP-1 and c-Myc pathways, thereby promoting oxidative stress in myocardial tissue and aggravating myocardial hypertrophy and ventricular remodelling (Ref. 67).

Epidermal growth factor receptor (EGFR) is a member of receptor tyrosine kinase whose deficiency could worsen myocardial hypertrophy, cardiac dysfunction and arterial hypotension (Refs 68, 69). A series of classic pro-hypertrophic factors, such as Ang II and norepinephrine, could bind to their cell membrane receptors and activate EGFR (Refs 70, 71). The intracellular tyrosine kinase domain of EGFR actively participates in signal transduction, activating the PI3K/Akt, MAPK and other signalling pathways. In addition, patients receiving EGFR tyrosine kinase treatment are particularly prone to cardiac complications (Ref. 72). Li *et al.* identified UCHL1 as a pathogenic factor of cardiac hypertrophy through microarray analysis. By recognizing and cutting the ubiquitin chains connected with EGFR, UCHL1 reduced the degradation of EGFR through the ubiquitin-proteasome system, thus continuously activating EGFR, Akt and ERK signalling pathways, leading to myocardial hypertrophy (Ref. 9). The cardiac dysfunction of mice with cardiac hypertrophy induced by TAC was dramatically relieved after treatment with LDN-57444, an inhibitor of UCHL1, indicating that UCHL1 may be employed as a potential drug target for the therapy of cardiac hypertrophy in the future.

Moreover, another study on the role and mechanism of UCHL1 in cardiac hypertrophy induced by pressure overload revealed that the expression of UCHL1 was upregulated in hypertrophic heart, while the overexpression of UCHL1 could inhibit the growth of fibroblasts, and the inhibition of growth was not related to apoptosis. Interestingly, other research has unveiled that UCHL1 could enhance the proliferation of a range of cells, such as HeLa cells, Neuro2a cells, human cancer cell lines h727 and MCF (Ref. 73). Based on the conclusion of the above research, investigators confirmed that the inhibitory effect of UCHL1 on cell growth inhibition is unique to cardiac fibroblasts. The inhibitory effect was caused by affecting the level of cyclin-dependent kinase inhibitor protein p21^WAF1/Cip1^ instead of affecting the signal pathway related to fibroblast proliferation. Interestingly, further experiments of the team showed that UCHL1 increased the protein content of p21^WAF1/Cip1^ by inhibiting the autophagy lysosomal degradation of p21 rather than by inhibiting the ubiquitin-proteasome system (Ref. 74).

Deubiquitinases also exert regulatory effects on the pathogenesis of heart failure-related stroke. Researchers analysed and compared heart failure-related chip data and stroke-related chip data through bioinformatics analysis. OTU deubiquitinase with linear linkage specificity (OTULIN) was identified as a regulator in the pathogenesis of heart failure-related stroke through the intersection analysis of heart failure-related chip data and stroke-related chip data. It may participate in the regulation of heart failure-related stroke by participating in protein ubiquitination and Wnt signalling. OTULIN is expected to become a key regulator in the pathophysiological process of heart failure-related stroke (Ref. 75).

Deubiquitinases are also involved in the pathogenesis of right ventricular hypertrophy (RVH). The researchers indicated that the expression of UCHL1 was evaluated in the RVH model induced by pulmonary artery ligation (PAC). However, whether UCHL1 is involved in the pathogenesis of RVH and its molecular mechanism is not yet clear, and additional research is warranted to clarify the role of UCHL1 in RVH (Ref. 76).

### Myocardial infarction (MI) and myocardial ischaemia reperfusion (I/R) injury

MI is driven by the imbalance between myocardial oxygen supply and demand, which results in myocardial necrosis. Researchers discovered that the mRNA expression of USP19 was downregulated in the acute model of MI, which raised the possibility that USP19 could have a special function in MI (Ref. 36). However, the mechanism of USP19 in MI remains unknown.

Apoptosis is a key process in the pathogenesis of MI and cardiac dysfunction. ABRO1 is also one of the four subunits (ABRO1, NBA1, BRE and BRCC36 proteins) of deubiquitinase BRISC. According to the research, the hearts of MI patients were characterized by higher protein levels of ABRO1 protein. The protein level ABRO1 was upregulated in the heart of MI mice and the knockdown of ABRO1 worsened myocardial cell apoptosis. However, it is unclear how ABRO1 specifically prevents cardiomyocytes from cell apoptosis (Ref. 77). Besides, study has shown that the expression of USP7 was upregulated in H9C2 cardiomyocytes cultured under hypoxia and in the heart of MI rats, and overexpression of USP7-induced inflammation and apoptosis of cardiomyocytes, leading to aggravation of MI injury. The study further pointed out that the upregulation of USP7 in the MI model was partly due to the downregulation of miR-409-5p (Ref. 78). Other researchers detected the expression of USP6 and USP47 from monocytes isolated from peripheral blood samples of MI patients and healthy controls. The expression of USP6 and USP47 was upregulated, especially the level of USP47. The team further found that USP47 may enhance the activity of the NF-*κ*B promoter, activating the NK-*κ*B pathway, thereby promoting cardiomyocyte apoptosis and I/R injury (Ref. 79).

Ferroptosis is a kind of programmed cell death that depends on iron, which is different from apoptosis and necrosis. Inhibition of ferroptosis can effectively reduce myocardial injury caused by ischaemia-reperfusion (Ref. 80). The expression of USP7 was evaluated in the myocardial tissue of rats with ischaemia-reperfusion injury. Knockdown of USP7 could inhibit ferroptosis by deubiquitinased p53. The deficiency of USP7 reduced MI area and alleviated myocardial fibrosis through the inhibition of p53/TfR1 pathway (Ref. 81). In addition to the deubiquitinase mentioned above, recent study has shown that OTUD5 played an important role in the mechanism of ferroptosis exacerbating myocardial ischaemia-reperfusion injury. OTUD5 inhibited ferroptosis and alleviated myocardial injury by directly binding to glutathione peroxidase 4 (GPX4), the key protein of ferroptosis, and removing the K48-linked ubiquitin chains of GPX4 (Ref. 82).

Deubiquitinase can not only regulate myocardial cell apoptosis and ferroptosis, but also modulate myocardial cell pyroptosis. Recent study has shown that USP11 promoted cardiomyocyte pyroptosis and exacerbated myocardial ischaemia-reperfusion injury by binding to TRAF3 protein and hydrolysing its ubiquitin chains (Ref. 83).

In addition to the programmed cell death, inflammatory cells and inflammatory factors are also important mechanisms for the occurrence and development of MI. As a cytokine combined with ST2, IL-33 plays a protective role in acute MI by reducing inflammation and apoptosis. It was reported that USP17 maintained the stability of IL-33 by cleaving and hydrolysing both the K48- and K63-linked ubiquitin chains (Ref. 84). In the heart of AMI mice, inhibition of lncRNA ANRIL weakened the degradation of IL-33 mediated by USP17. Reduction of degradation of IIL33 through the ubiquitin proteasome system alleviates cardiac dysfunction and cardiac fibrosis by reducing infarct size and inhibits cell apoptosis (Ref. 85).

Previous studies have shown that dual specificity phosphatase 1 (DUSP1)-mediated JNK dephosphorylation participated in protecting the heart from ischaemia-reperfusion injury through the anti-apoptotic effect (Ref. 86). According to recent research, USP49 has been confirmed as a regulator of DUSP1-JNK1/2 signal transductions. The expression of USP49 was upregulated in the I/R injury model on AC16 cardiomyocytes, and the signal transduction pathway of DUSP1-JNK1/2 was activated to exert its anti-apoptotic role (Ref. 87).

After myocardial ischaemia-reperfusion injury, myocardial cell apoptosis, autophagy and immune cell infiltration prompt the imbalance of a series of cytokines, eventually activating cardiac fibroblasts and leading to myocardial fibrosis (Ref. 88). TGF*β*/Smad4 signalling pathway is identified as a classic pro-fibrosis pathway and is modulated by USP10 (Ref. 89). It is demonstrated that, in fibroblasts, HSP47 aggravated chronic fibrosis after myocardial ischaemia-reperfusion by recruiting USP10, which removed the ubiquitin chains of Smad4, and stabilized Smad4 protein (Ref. 90).

In addition, under pathological conditions such as the stimulation of TGF-*β*, myocardial fibroblasts undergo glycometabolic reprogramming, mainly manifested as enhanced glycolysis (Refs 91, 92, 93). Inhibiting glycolysis could alleviate myocardial fibrosis. Researchers found that the protein level of PFKFB3, a sort of glycolytic enzyme, was upregulated by the deubiquitinase OTUD4 in myocardial fibroblasts of post MI mice. OTUD4 promoted the stability of PFKFB3, leading to enhanced glycolysis and exacerbating cardiac fibrosis (Ref. 94).

Based on previous studies, sevoflurane postconditioning has been considered as a potential treatment to protect the myocardium from I/R injury by limiting the size of MI, reversing myocardial dysfunction, and improving blood circulation (Ref. 95). Researchers found that the expression of USP22 was upregulated in the hearts of I/R mice treated with sevoflurane postconditioning, and knockdown of USP22 could reverse the protective effect of sevoflurane. Sevoflurane postconditioning upregulated USP22, which increased the protein content of lysine-specific demethylase 3A (KDM3a) in the promoter region of YAP1 by cutting the ubiquitin chains of the protein. The upregulation of USP22 indirectly promoted the transcription of YAP1, thereby reduced myocardial cell injury (Ref. 96).

**Figure 2.**
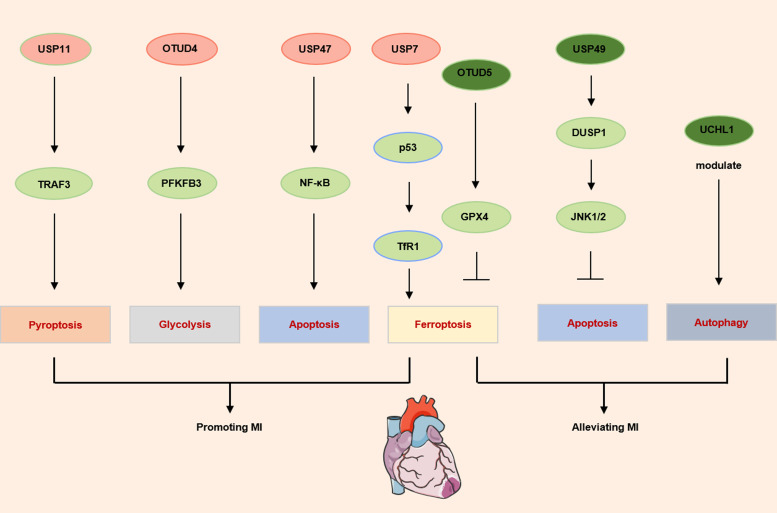
The main deubiquitinases involved in the development of myocardial infarction. *Notes*: A series of deubiquitinases regulate myocardial infarction through apoptosis, ferroptosis, pyroptosis or metabolic reprogramming. USP: ubiquitin-specific protease; TRAF3: TNF receptor-associated factor 3; PFKFB3: 6-phosphofructo-2-kinase/fructose-2,6-bisphosphatase 3; OTUD: ovarian tumor-related deubiquitinase; NF-κB: nuclear factor kappa-B; TfR1: transferrin receptor 1; GPX4: glutathione peroxidase 4; DUSP1: dual specificity phosphatase 1; JNK1/2: c-Jun N-terminal kinase 1/2; UCHL1: ubiquitin C-terminal hydrolases L1.

Besides, appropriate level of autophagy in cardiomyocytes is considered as a protective mechanism determining the cardiac remodelling. Excessive activation or inhibition of autophagy could deteriorate MI (Refs 97, 98). Research has shown that deficiency of UCHL1 in the myocardium after MI could exacerbate cardiac remodelling by inhibiting autophagy and decreasing autophagic flux. The research further revealed that the upregulation of autophagic flux was induced by the abnormal formation of autophagosome instead of the increased degradation of autophagosomes. Interestingly, this research pointed out that the absence of UCHL1 in cardiomyocytes has little impact on heart development. No abnormalities were observed in the cardiac development of UCHL1-specific knockout mice before 14 weeks of age (Ref. 99) (Fig. 2).

### Diabetes mellitus (DM) related cardiac disease

Diabetic heart disease is characterized by dysregulated cardiac structure and function that is independent of diabetic macrovascular complications (including hypertension, coronary artery disease and atherosclerosis) (Ref. 100). Mitochondria are important integrators of redox signal and metabolic flux, and mitochondria dysfunction is involved in the occurrence and development of diabetic heart disease (Ref. 101). S-sulfhydrylation is a new post-translational modification of specific cysteine residues on target proteins by H_2_S (Ref. 102). A study has shown that under the conditions of high glucose and high fat in vivo and in vitro, the S-sulfhydrylation of USP8 was significantly reduced, while after exogenous H_2_S treatment, the S-sulfhydrylation level of USP8 was increased, promoting the combination of USP8 and Parkin. The combination of USP8 and Parkin ameliorated cardiac dysfunction in mice with type2 diabetes mellitus (T2DM) by regulating mitochondrial autophagy, reducing mitochondrial fusion and improving cell mitochondrial function (Ref. 103). The deubiquitinase-regulated downstream process involved not only mitochondrial autophagy, but also the abundance alterations of transcription factors. Y-box binding protein-1 (YB-1), as a regulatory factor for transcription and translation, protects cardiomyocytes from apoptosis and ameliorates cardiac fibrosis (Ref. 104). YB-1 is regulated by various post-translational modifications, including ubiquitination (Ref. 105). Study in vitro and in vivo indicated that hyperglycaemia caused the phosphorylation of S102 site of YB-1 in cardiomyocytes, which weakened the interaction between YB-1 and OTUB1, thus promoting the degradation of YB-1 through ubiquitin-proteasome pathway, leading to aggravation of diabetes cardiomyopathy (Ref. 106).

Compared with the general population, patients with T2DM are more likely to suffer from MI, which is a multi-step event characterized by myocardial fibrosis, cardiomyocyte apoptosis and cardiac dysfunction (Ref. 107). Follicle-like protein 1 (FSTL1) is a glycosylated secretory protein produced by mesenchymal cell lines such as cardiomyocytes and fibroblasts. It is an acidic secretory protein rich in cysteine and is considered to be a beneficial regulator of cardiac fibrosis and insulin resistance. Recent studies have confirmed that the protective effect of FSTL1 in T2DM with MI was mediated by the USP10/Notch1 axis. Moreover, the inhibition of the USP10/Notch1 axis could counteract the myocardial protection of FSTL1 in T2DM (Ref. 108).

### Myocardial disease

Myocarditis is a local or diffuse inflammatory lesion of the heart characterized by inflammatory cell infiltration, cardiomyocyte degeneration and necrosis (Ref. 109). In coxsackie virus B3 (CVB3)-infected myocarditis, the expression of miR-21 that targeted the mRNA of the deubiquitinase YOD1 was up-regulated, resulting in the downregulation of YOD1. The decreased expression of YOD1 led to the increase of the K48-linked ubiquitin chains of desmin protein, causing the degradation of desmin through the ubiquitin proteasome pathway. The degradation of desmin induced by YOD1 resulted in desmosome damage and disc dysfunction (Ref. 110).

Dilated cardiomyopathy (DCM) is a non-ischaemic cardiomyopathy with left ventricular dilation and systolic dysfunction, without coronary artery disease or abnormal load proportional to the degree of left ventricular damage (Ref. 111). By collecting myocardial samples from patients with DCM and volunteers in the control group, the researchers found that the protein level of p53 in patients with DCM was associated with the upregulation of herpesvirus-associated ubiquitin specific protease (HAUSP). HAUSP stabilized p53 by removing ubiquitin chains, resulting in increased cardiomyocyte apoptosis (Ref. 112).

In DOX-induced cardiomyopathy, USP19 was downregulated in cardiomyocytes, which accelerated the degradation of TRAF2. The decrease in TRAF2 protein level led to abnormal NF-*κ*B signalling, resulting in mitochondrial abnormalities and cell necrosis (Ref. 113).

### Rheumatic heart disease

Rheumatic heart disease (RHD) is a valvular heart disease caused by acute rheumatic fever (Ref. 114). It is an autoimmune sequela of suppurative streptococcal mucosal infection (Ref. 115). As one of the CD4^+^ T cell subsets, Th17 secretes IL-17 to mediate inflammation as well as autoimmunity and promote inflammatory response as well as disease progression in patients with RHD. By isolating T cells from patients with RHD as well as healthy volunteers and detecting the mRNA level of USP4 in CD4^+^ T cells, the researchers found that the mRNA level of USP4 was related to the mRNA level of IL-17 and the function of Th17, indicating that USP4 may mediate the function of Th17 under inflammatory stimulation. The preliminary study of USP4 provided experimental evidence that USP4 may be a future therapeutic target for RHD (Ref. 116).

## Deubiquitinase inhibitors associated with cardiac disease

There are approximately 30 types of deubiquitinase inhibitors (Ref. 19), and most of the research on deubiquitinase inhibitors focuses on tumours (Refs 18, 117). In terms of heart disease, a recent study revealed that UCHL1 inhibitors inhibited a variety of signalling pathways related to myocardial hypertrophy and fibrosis, such as AKT, ERK1/2, STAT3, calcineurin A, TGF-*β*/Smad2/3 and NF-*κ*B, which alleviated hypertension-induced myocardial hypertrophy and fibrosis (Ref. 118). P22077 is the inhibitor of USP7 with the protective role in cardiac hypertrophy and cardiac remodelling. The deubiquitinase inhibitor repressed multiple signalling pathways such as AKT/ERK and TGF-*β*/SMAD2/collagen I/collagen III, NF-*κ*B/NLRP3 and NAPDH oxides, leading to the inhibition of inflammation and oxidative stress (Ref. 119).

As is exhibited in Table 1, there is little research on the role of deubiquitinase inhibitors in cardiac disease, especially in cardiac hypertrophy, and the vast majority of deubiquitinase inhibitors are still in the pre-clinical stage, with no clinical research on deubiquitinase in cardiac disease (Ref. 18). Compared with other deubiquitinase inhibitor, the anti-hypertrophic role of LDN-57444, the inhibitor of UCHL1, seems more explicit. LDN-57444 may serve as a new therapeutic direction in the future.

**Table 1.** The function and mechanism of deubiquitinase involved in regulating cardiac disease

Inhibitor	Target deubiquitinase	Disease	Function	Mechanism	Reference
LDN-57444	UCHL1	Cardiac hypertrophy	Alleviate cardiac hypertrophy	Reduce the stability of EGFR	11
	UHCL1		Alleviate hypertension-induced cardiac hypertrophy	Inhibit the activation of AKT, ERK1/2, STAT3, calcineurin A, TGF-*β*/Smad2/3 and NF-*κ*B	118
P22077	USP7		Alleviate cardiac hypertrophy and cardiac remodelling	Inhibit the activation of AKT/ERK and TGF-*β*/SMAD2/collagen I/collagen III, NF-*κ*B/NLRP3 and NAPDH oxides	119
IU1	USP14		Alleviate cardiac hypertrophy	Decrease GSK-3*β* phosphorylation	48
Spautin-1	USP10	T2DM-MI	Accelerate T2DM-associated MI	Inhibit USP10/Notch1 signalling	108

Several studies have shown that the accumulation of ubiquitinated proteins induced by deubiquitinase can lead to endoplasmic reticulum (ER) stress and activation of autophagy (Refs 120, 121). Both broad-spectrum inhibitors of deubiquitinases and specific inhibitors of individual deubiquitinases can lead to an increase in the ubiquitination of total proteins, thereby activating ER stress, unfolded protein response and AMPK pathway-mediated autophagy (Refs 122, 123). In hepatocellular carcinoma, the deubiquitinase inhibitor bAP15 (a specific inhibitor of UCHL5 and USP14) or ML-323 (a specific inhibitor of USP1) could increase the ubiquitination level of total proteins and activate ER stress, thereby inhibiting the viability and migration of cancer cells (Refs 124, 125). However, currently, there is few relevant research focusing on the relationship between deubiquitinase inhibitors and ER stress and ER stress-related autophagy in cardiac disease. In the future, more research is needed to focus on the potential impact of deubiquitinase inhibitors on ER stress and its related autophagy in cardiac disease.

## Deubiquitinase functions beyond the cardiac disease

Deubiquitinases not only participate in regulating cardiac disease but also play an indispensable role in other diseases, including vascular disease, cancer and neurodegenerative diseases (Refs 25, 126, 127). Among the vascular diseases that are closely related to cardiac disease, deubiquitinases have been proven to be related to the onset and disease progression of atherosclerosis, vascular calcification, aneurysm onset and disease progression (Ref. 25). Furthermore, the emerging role of deubiquitinases in cancer is also a research hotspot. It is demonstrated that deubiquitinase USP25 promoted tumour growth of pancreatic cancer in conversion with HIF-1*α* (Ref. 128). Taken together, deubiquitinases target a variety of proteins, endowing them with the ability to regulate physiological and pathological processes such as eukaryotic cell division, differentiation and apoptosis. Further exploration of the role of deubiquitinases in cardiac disease is promising.

## Conclusion

Deubiquitinases can recognize a variety of proteins related to the progression and pathophysiological process of cardiac disease, stabilize the protein by removing the ubiquitin chains, then affect the activation of the signal pathway that is downstream of the substrate protein, and finally regulate the basic biological processes such as inflammation, apoptosis and autophagy. According to an increasing number of studies, deubiquitinases were implicated in the regulation of the pathophysiology progression of cardiac disease, and its abundance changes in a variety of pathological stimuli, which then affects the post-translational modification of proteins in vivo. However, there are still many unknowns about the mechanism of deubiquitinases regulating cardiac disease that needs to be further explored. There is still a large span from animal experiments to human research. A deeper study of the mechanism on the role of deubiquitinases and the study of related inhibitors will contribute to providing new therapeutic targets for cardiac disease in the future.
